# White blood cell DNA methylation and risk of breast cancer in the Prostate, Lung, Colorectal, and Ovarian Cancer Screening Trial (PLCO)

**DOI:** 10.1186/s13058-017-0886-6

**Published:** 2017-08-18

**Authors:** Susan R. Sturgeon, J. Richard Pilsner, Kathleen F. Arcaro, Kaoru Ikuma, Haotian Wu, Soon-Mi Kim, Nayha Chopra-Tandon, Adam R. Karpf, Regina G. Ziegler, Catherine Schairer, Raji Balasubramanian, David A. Reckhow

**Affiliations:** 1 Department of Biostatistics and Epidemiology, University of Massachusetts-Amherst, 715 North Pleasant Street, Arnold House 407, Amherst, MA 01003 USA; 2 Department of Environmental Health Sciences, University of Massachusetts, Amherst, MA 01003 USA; 3 Department of Veterinary and Animal Sciences, University of Massachusetts, Amherst, MA 01003 USA; 4 Department of Civil and Environmental Engineering, University of Massachusetts, Amherst, MA 01003 USA; 5 Department of Civil, Construction and Environmental Engineering, Iowa State University, Ames, IA 50011 USA; 6 Eppley Institute and Fred & Pamela Buffett Cancer Center, University of Nebraska Medical Center, Omaha, NE 68198 USA; 7 Epidemiology and Biostatistics Program, Division of Cancer Epidemiology and Genetics, National Cancer institute, Bethesda, MD 20892 USA

**Keywords:** Breast cancer, White blood cells, Global DNA methylation, %5mdC, Cohort

## Abstract

**Background:**

Several studies have suggested that global DNA methylation in circulating white blood cells (WBC) is associated with breast cancer risk.

**Methods:**

To address conflicting results and concerns that the findings for WBC DNA methylation in some prior studies may reflect disease effects, we evaluated the relationship between global levels of WBC DNA methylation in white blood cells and breast cancer risk in a case-control study nested within the Prostate, Lung, Colorectal and Ovarian Cancer Screening Trial (PLCO) cohort. A total of 428 invasive breast cancer cases and 419 controls, frequency matched on age at entry (55–59, 60–64, 65–69, ≥70 years), year of entry (on/before September 30, 1997, on/after October 1, 1997) and period of DNA extraction (previously extracted, newly extracted) were included. The ratio of 5-methyl-2’ deoxycytidine [5-mdC] to 2’-deoxyguanine [dG], assuming [dG] = [5-mdC] + [2’-deoxycytidine [dC]] (%5-mdC), was determined by liquid chromatography-electrospray ionization-tandem mass spectrometry, an especially accurate method for assessing total genomic DNA methylation.

**Results:**

Odds ratio (OR) estimates and 95% confidence intervals (CI) for breast cancer risk adjusted for age at entry, year of entry, and period of DNA extraction, were 1.0 (referent), 0.89 (95% CI, 0.6–1.3), 0.88 (95% CI, 0.6–1.3), and 0.84 (95% CI, 0.6–1.2) for women in the highest compared to lowest quartile levels of %5md-C (*p* for trend = .39). Effects did not meaningfully vary by time elapsed from WBC collection to diagnosis.

**Discussion:**

These results do not support the hypothesis that global DNA hypomethylation in WBC DNA is associated with increased breast cancer risk prior to the appearance of clinical disease.

## Background

Cytosines can be methylated in the mammalian genome, primarily at CpG sites. CpG sites are located throughout the genome, including gene promoter regions and repetitive DNA sequences. DNA methylation patterns play a key role in gene expression and cell integrity. For example, genome-wide hypomethylation can be associated with chromosomal instability and the expression of oncogenes or repetitive sequences that are normally silenced by methylation [1]. Global loss of DNA methylation is characteristic of cancer tissue [2].

There are several different methods to assess global DNA methylation. Measurement of the ratio of 5-methyl-2’ deoxycytidine [5-mdC] to 2’-deoxyguanine [dG], assuming [dG] = [5-mdC] + [2’-deoxycytidine [dC]] (%5-mdC), by liquid chromatography-electrospray ionization-tandem mass spectrometry (LC-ESI-MS/MS) provides a comprehensive measure of genome-wide DNA methylation levels [3]. This method is considered the gold standard as it evaluates the entire genome but is expensive and time-consuming, and requires specialized laboratory equipment. Approximately one-third of the DNA methylation in the genome occurs in the repetitive sequences of the genome, including *LINE*-*1* and *Alu* [4]. For this reason, DNA methylation levels in *LINE*-*1* or *Alu* repeats, which can be obtained by high-throughput methods, have often been used as surrogate markers of global DNA methylation [5]. Other high-throughput surrogate methods for estimating global methylation are available. For example, the luminometric methylation assay (LUMA) [6] estimates global DNA methylation levels by using restriction enzymes specific for methylated and unmethylated CCGG, a sequence found throughout the genome. A different approach has been to average methylation levels across the limited set of individual CpG sites represented on the Illumina human methylation bead kits [7, 8]. An advantage of this latter approach, relative to aggregrate methods of assessing global methylation (e.g., %5mdC, LINE-1), is that one can adjust for potential differences in blood cell composition between cases and controls in archived blood specimens [9]. A second advantage is that it is possible to conduct subanalyses to examine methylation in specific locations in the genome (e.g., promoter regions across the genome) [7, 8].

Global DNA hypomethylation of breast tumor tissue is well-established [2]; however, there is some evidence that global hypomethylation in circulating white blood cell (WBC) DNA may also be associated with an increased risk of breast cancer. One possible explanation is that the association represents unidentified environmental and lifestyle determinants that influence both global methylation and breast cancer risk. An alternative possibility is that, in response to very early, preclinical breast cancer, a new clone of circulating leukocytes arises that alters white blood cell DNA methylation [10]. In a relatively small retrospective case-control study, Choi and colleagues [11] observed a nearly threefold increase in breast cancer risk among women in the lowest tertile of %5-mdC in WBC DNA compared to women in the highest tertile. Comparable results were obtained prospectively in the case-cohort study nested in the NIEHS Sister Cohort Study [12], with a nearly twofold increased risk observed among women in the lowest quartile of *LINE*-*1* WBC DNA methylation compared with those in the highest quartile. By contrast, a number of other investigations, including three separate nested case-control studies from Europe, which used pre-diagnostic DNA (and were presented in a single publication) [13], observed no association between *LINE*-*1* methylation in WBC DNA and breast cancer risk [11, 13–18] or between *Alu* methylation in WBC DNA and breast cancer risk [14, 16, 19]. Findings from the three retrospective case-control studies employing the LUMA assay were inconsistent, with positive, inverse, and null associations [15, 20, 21]. However, three of the four nested case-control studies, which used the Illumina HumanMethylation450 BeadChip on prediagnostic DNA (separate results from three cohorts were reported in a single paper [8]) observed that global hypomethylation was positively associated with increased breast cancer risk [7, 8].

To address these discrepant results, we examined the association between global hypomethylation in WBC DNA and subsequent breast cancer incidence in a study nested in the Prostate, Lung, Colorectal and Ovarian Cancer Screening (PLCO) Trial cohort. We measured WBC DNA %5-mdC levels by LC-ESI-MS/MS because: (1) WBC global hypomethylation as measured by %5-mdC level was found to be strongly and significantly associated with increased breast cancer risk in the one breast cancer study that measured %5-mdC, which was a relatively small restrospective study [11]; (2) global hypomethylation as measured by WBC DNA %5-mdC was reported to be more strongly associated with overall cancer risk than surrogate measures of global methylation (*LINE*-*1*, *Alu*, and LUMA) in a recent meta-analysis [22]; and (3) %5-mdC level measured by LC-ESI-MS/MS is considered the gold standard assay for accurately assessing methylation across the entire genome.

Our study is important because it is the first investigation to examine the association between WBC DNA %5-mdC levels, measured prior to breast cancer diagnosis, and subsequent breast cancer incidence. In this large study of 428 cases and 419 controls, the elapsed time between blood collection and breast cancer development ranged from 1.0 to 9.5 years, enabling us to examine whether risk varied by time elapsed between blood collection and diagnosis.

## Methods

### Selection of study subjects

Cases and controls for the present analysis were selected from the Etiology and Early Marker Study (EEMS) breast cancer case-control study that was established from the 39,115 women randomized to the intervention arm of the PLCO screening trial [23]. Through June 30, 2005, a total of 1141 eligible cases of breast cancer were identified. A total of 1141 controls were frequency matched to cases by randomly sub-sampling women who had not been diagnosed with breast cancer by June 30, 2005 in eight strata defined by four age categories (55–59, 60–64, 65–69, ≥70 years) and time of entry into the study (on/before September 30, 1997, on/after October 1, 1997).

A total of 732 cases and 928 controls were initially identified as eligible for the present analysis, after further excluding in the following order: subjects who did not give permission for genetic studies (cases = 32, controls = 25), subjects who had a personal history of any cancer prior to the trial (cases = 40, controls = 30), subjects with unconfirmed, erroneous or in situ breast cancer (cases = 229, controls = 13), subjects who developed other types of cancer anytime during follow up (cases = 105, controls = 130), and other reasons (cases = 3, controls = 14).

A total of 649 out of the 726 cases and 787 out of the 928 controls either had: (1) DNA already extracted from buffy coat/whole blood remaining from a prior study; or (2) buffy coat available for extraction. We further excluded 83 breast cancer cases in which diagnosis occurred within one year of the DNA collection, to minimize the likelihood of disease effects, leaving an eligible pool of 566 cases and 787 controls.

For efficiency, our a priori plan was to select 430 cases and 430 controls for this analysis. We first prioritized case selection to include the 151 cases and 147 controls that already had DNA extracted as part of another study [24]. We then supplemented study subject selection to include cases and controls with buffy coat available for DNA extraction for this study. Controls were frequency matched to cases on age, calendar year of entry, and the date of the DNA extraction (already extracted DNA or newly extracted DNA). Matching on date of DNA extraction was done to address the possible concern that DNA methylation patterns may be affected by the timing or method of DNA extraction. We ultimately selected 428 breast cancer cases and 420 controls that had suitable DNA for analysis, slightly less than our goal because some of the subjects we originally selected turned out to have inadequate DNA. One additional control subject was later excluded for an improbable value for %5-mdC (67.4%). Thus, our final analysis consisted of 428 cases and 419 controls. The institutional review boards of the National Cancer Institute, the 10 participating study centers, and the University of Massachusetts Amherst approved this study. Informed consent was obtained from all participants at study enrollment.

### DNA extraction

For 298 study subjects, DNA was previously extracted in 2006–2007 from stored buffy coat (98%) or whole blood (2%). About 90% of this DNA was extracted using the Autopure method (Qiagen) and the remaining specimens were extracted using standard phenol/chloroform extraction. In 2014, DNA was extracted for the remaining study subjects using the QIAsymphony SP automated extraction robot (Qiagen). DNA concentrations were quantified using the Picogreen assay and nanodrop technology.

### DNA hydrolysis

To provide individual nucleosides for subsequent total methylated cytosine measurements, genomic DNA were hydrolyzed with DNA Degradase Plus (Zymo Research, Cat # E2021) following the manufacturer’s protocol with minor adjustments. Briefly, 400 ng of genomic DNA was incubated with 5 U of DNA Degradase Plus in 25 μl total reaction volumes at 37 °C for 2 hours. Batch control DNA included female genomic DNA (Promega, Cat # G1521), which was considered to be “normally” methylated. Complete DNA hydrolysis of additional control samples were verified by agarose gel electrophoresis. Hydrolyzed DNA samples were stored at −20 °C until %5-mdC analyses.

### Measurement of %5-mdC

The %5-mdC levels were determined by LC-ESI-MS/MS after hydrolysis of DNA as described by Song and colleagues [3], with modifications. Both 5-mdC and dG concentrations were quantified with internal standard additions of isotope-labeled 5-mdC and dG (5-mdC-d3 and 15 N5-dG, respectively; obtained from Toronto Research Chemicals). LC separation was performed on an Acquity UPLC system (Waters Corporation) at a flow rate of 400 μL/min. Methanol containing 0.1% formic acid and water containing 0.1% formic acid were used as buffers. The organic buffer ratio was increased at a linear gradient from 0 to 22.5% over 8 min for the elution of nucleosides. The sample injection volume was 20 μL. Detection by ESI-MS/MS was performed on a Quattro Premier XE Mass Spectrometer (Waters Corporation) following LC separation. The following optimized conditions for ESI positive ion mode were used: source temperature, 120 °C; desolvation gas flow, 700 L/h; cone gas flow, 50 L/h; capillary voltage, 4.2 kV; cone voltage, 10 V; extractor voltage, 2 V; entrance potential voltage, 0 V; collision energy, 11 V; and collision cell exit potential, 2.0 V. Multiple reaction monitoring mode was utilized for the quantification of native and labeled nucleosides. The transition pairs of molecular and fragment ions monitored were m/z 242.0/126.0 for 5-mdC, m/z 245.0/129.0 for 5-mdC-d3, m/z 268.1/152.0 for dG, and m/z 273.0/157.0 for 15 N5-dG with a scan time of 150 minutes for each pair. Following QuanLynx (Waters Corporation) analysis for chromatographic peak detection, the resulting peak areas of the native nucleosides were normalized to the labeled internal standards and quantified based on external calibration curves. The 5-mdC and dG nucleosides for external calibration were obtained from Fisher Scientific. We took the average of two injections from each sample vial. Results are reported as the ratio between 5-mdC and dG, assuming that [dG] = [5-mdC] + [dC]. Laboratory personnel were blinded to case status.

Samples were run on twelve plates, with cases and controls distributed on each plate approximately evenly within eight stratum defined by age and time of entry. Each plate had DNA that was either all pre-extracted or all newly extracted. On each plate, we also included three replicate DNA specimens from three women who were in a study site for the PLCO trial that was later dropped (newly extracted DNA). The mean inter-batch coefficient of variation across the twelve plates for each of the three women was 8, 7, and 9%, respectively. The female genomic DNA average inter-batch coefficient of variation was comparable at 9.2%.

**Fig. 1:**
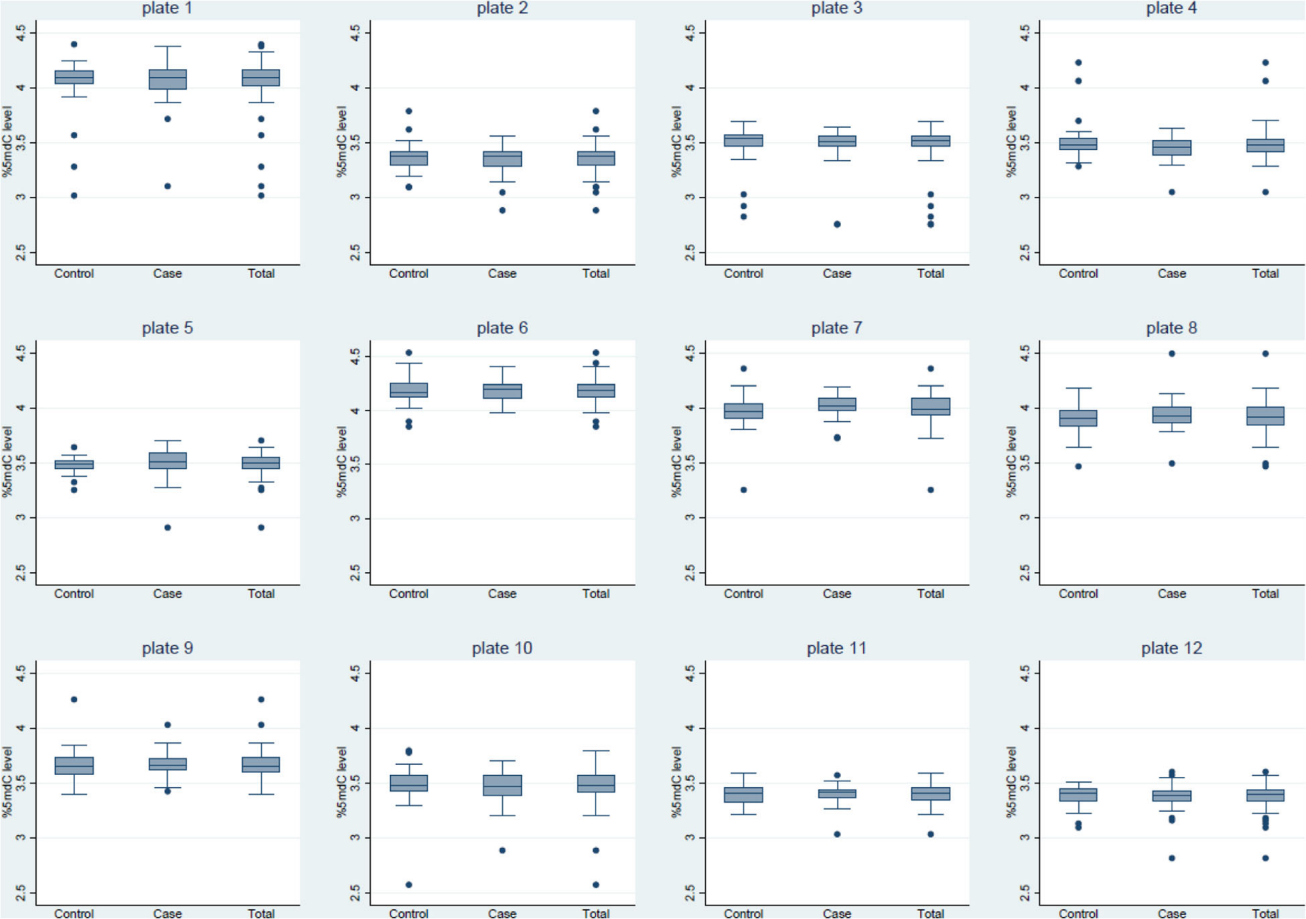
Boxplot distribution of the ratio of 5-methyl-2’ deoxycytidine (5-mdC) to 2’-deoxyguanine (dG) (%*5*-*mdC*) levels by case-control status in each of twelve laboratory plates. The *ends* of the boxes are the 25^th^ and 75^th^ percentiles and the *center line* is the median. The *lengths* of the *whiskers* are defined as 1.5*(75^th^ percentile – 25^th^ percentile). The *circles* are values outside the whisker length

In Fig. 1, we show levels of %5-mdC for study subjects in each of the twelve batches and also separately for cases and controls in each batch. Levels of %5-mdC varied by batch and showed limited variation across individuals within batch.

#### Breast cancer ascertainment

Breast and other cancers were primarily identified through an annual study update mailed to participants, which established cancer diagnosis in the previous year, including type and date [25]. Non-respondents were contacted by mail and telephone. In order to confirm the self-reported cancers, medical records (for standardized medical record abstraction of pathology reports) were retrieved and usually obtained within 2 years of self-report. Cancers were also identified through death certificates, data obtained from state cancer registries, and information from next-of-kin for deceased participants.

#### Ascertainment of other variables

Demographic information, medical history, and health-related behavior were obtained through baseline questionnaires completed by study participants at or around the time of randomization.

#### Statistical analysis

Logistic regression was used to estimate odds ratios and 95% CI, for breast cancer with case/control status as the outcome and a categorical variable denoting quartiles of the %5-mdC levels as the primary exposure of interest. Because of the evidence of inter-batch variation, we created %5-mdc quartile levels separately for each batch and then created a new summary variable with four levels based on the batch-specific cut points. For example, individuals in the lowest category of the summary variable included individuals from each of the twelve batches who were placed in the lowest batch-specific quartile ranking. Models were adjusted for matching variables, including age in four categories (55–59, 60–64, 65–69, ≥70 years), time of entry into the study (on/before September 30, 1997, on/after October 1, 1997), and period of DNA extraction (previously extracted, newly extracted). Additional models considered adjustment for established or suspected breast cancer risk factors, including race, body mass index, age at menarche, age at first live birth, number of children, type of menopause, age at natural menopause, personal history of benign breast disease, cigarette smoking, recent alcohol intake, family history of breast cancer, and menopausal hormone use. Statistical significance was assessed for each level of the primary exposure variable using two-sided Wald hypothesis tests.

## Results

Relative risks for established breast cancer risk factors were generally comparable in our analytic subgroup of cases and controls to those reported previously in the literature [25] (Table 1). Nulliparity, later age at first birth, late age at natural menopause, a personal history of benign breast disease, a family history of breast cancer, and alcohol consumption were associated with increased breast cancer risk. Late age at menarche, three or more live births and surgical menopause were associated with reduced breast cancer risk.

**Table 1:** Associations between demographic and lifestyle characteristics and risk of breast cancer, adjusted for age and time of entry into the cohort

	Controls	%	Cases	%	Odds Ratio, 95% CI		
Age (years)							
<59	150	35.8	151	35.3	1.0 (referent)		
60–64	140	33.4	141	32.9	1.00	0.72	1.39
65–69	93	22.2	102	23.8	1.09	0.76	1.56
> =70	36	8.6	34	7.9	0.94	0.56	1.58
Time of entry							
> =October 10, 1997	168	40.1	174	40.7	1.0 (referent)		
< =September 30, 1997	251	59.9	254	59.4	1.02	0.78	1.35
Race							
White, non-Hispanic	380	90.7	395	92.3	1.0 (referent)		
Other	39	9.3	33	7.7	0.81	0.50	1.32
Body mass index (kg/m^2^)							
Underweight-normal (15–24.99)	175	41.9	176	41.2	1.0 (referent)		
Overweight (25.0–29.99)	141	33.7	152	35.6	1.07	0.79	1.46
Obese (> = 30)	102	24.4	99	23.2	0.96	0.68	1.36
Age at menarche (years)							
<12	70	16.8	88	20.6	1.0 (referent)		
12–13	238	56.9	219	51.2	0.73	0.51	1.05
> =14	110	26.3	121	28.3	0.87	0.58	1.31
Number of live births							
None	31	7.4	48	11.2	1.0 (referent)		
1–2 births	123	29.4	147	34.4	0.77	0.46	1.29
3+ births	265	63.3	233	54.4	0.57	0.35	0.92
Age at first birth (years)							
<20	72	17.2	55	12.9	1.0 (referent)		
20–24	209	49.9	181	42.5	1.14	0.76	1.70
25–29	80	19.1	108	25.4	1.77	1.12	2.79
> =30	27	6.4	34	8.0	1.66	0.89	3.07
Nulliparous	31	7.4	48	11.3	2.03	1.14	3.59
Type of menopause							
Natural	253	60.4	283	66.1	1.0 (referent)		
Bilateral oophorectomy	49	11.7	29	6.8	0.52	0.31	0.85
Other	117	27.9	116	27.1	0.88	0.65	1.20
Age at natural menopause (years)							
<50	91	36.0	95	33.7	1.0 (referent)		
50–54	122	48.2	141	50.0	1.15	0.79	1.69
55+	40	15.8	46	16.3	1.15	0.69	1.93
History of benign breast disease							
No	285	69.9	261	61.9	1.0 (referent)		
Yes	123	30.2	161	38.2	1.43	1.07	1.91
Smoking status							
Never	257	61.3	229	53.5	1.0 (referent)		
Former	135	32.2	163	38.1	1.36	1.02	1.82
Current	27	6.4	36	8.4	1.51	0.89	2.56
Recent alcohol intake							
No	112	29.2	99	24.9	1.0 (referent)		
Yes	272	70.8	298	75.1	1.25	0.91	1.72
Family history of breast cancer							
No	353	85.9	340	81.0	1.0 (referent)		
Yes	58	14.1	80	19.1	1.43	0.99	2.07
Hormone user							
Never	111	26.7	102	23.9	1.0 (referent)		
Former	70	16.8	55	12.9	0.86	0.55	1.34
Current	235	56.5	269	63.2	1.26	0.91	1.75

The batch-standardized quartile distribution of %5-mdC was unrelated to the distribution of breast cancer risk factors or study design matching factors (Table 2).

**Table 2:** Baseline distribution of study design and lifestyle risk factors by quartile of DNA methylation^a^

	Q4 (high)		Q3		Q2		Q1 (low)		
	*N*	%	*N*	%	*N*	%	*N*	%	*p* value
Age at entry (years)									
<59	85	39.5	67	31.5	76	37.3	73	34.0	
60–64	66	30.7	79	37.1	60	29.4	76	35.4	
65–69	46	21.4	48	22.5	52	25.5	49	22.9	
> =70	18	8.4	19	8.9	16	7.8	17	7.9	
									0.75
Time at entry									
< =September 30,1997	134	62.3	125	58.7	114	55.9	132	61.4	
> =October 1, 1997	81	37.7	88	41.3	90	44.1	83	38.6	
									0.53
Date of DNA extraction									
Pre-extracted DNA	69	32.1	69	32.4	70	34.3	90	41.9	
Newly-extracted DNA	146	67.9	144	67.6	134	65.7	125	58.1	
									0.12
Race/ethnicity									
White, non-Hispanic	194	90.2	197	92.5	190	93.1	194	90.2	
Other	21	9.8	16	7.5	14	6.9	21	9.8	
									0.60
Body mass index (kg/m^2^)									
Underweight-normal (15–24.9)	77	35.8	84	39.4	98	48.3	92	43.0	
Overweight (25.0 − 29.9)	91	42.3	72	33.8	60	29.6	70	32.7	
Obese (> = 30)	47	21.9	57	26.8	45	22.2	52	24.3	
									0.09
Age at menarche (years)									
<12	37	17.3	44	20.7	31	15.2	46	21.4	
13	116	54.2	122	57.3	107	52.5	112	52.1	
> =14	61	28.5	47	22.1	66	32.5	57	26.5	
									0.26
Number of live births									
None	18	8.4	20	9.4	16	7.8	25	11.6	
1–2	66	30.7	65	30.5	66	32.4	73	34.0	
> =3	131	60.9	128	60.1	122	59.8	117	54.4	
									0.75
Age at first birth (years)									
<20	36	16.8	32	15.0	24	11.8	35	16.3	
20–24	101	47.2	94	44.1	100	49.3	95	44.2	
25–29	47	22.0	54	25.4	48	23.7	39	18.1	
> =30	12	5.6	13	6.1	15	7.4	21	9.8	
Nulliparous	18	8.4	20	9.4	16	7.9	25	11.6	
									0.56
Type of menopause									
Natural	129	60.0	136	63.9	126	61.8	145	67.4	
Bilateral oophorectomy	20	9.3	21	9.9	17	8.3	20	9.3	
Other	66	30.7	56	26.3	61	29.9	50	23.3	
									0.67
Age at natural menopause (years)									
<50	53	41.1	46	33.8	41	32.8	46	31.7	
50–54	56	43.4	69	50.7	66	52.8	72	49.7	
55+	20	15.5	21	15.4	18	14.4	27	18.6	
									0.65
History of benign breast disease									
No	133	62.7	143	69.4	130	64.4	140	66.7	
Yes	79	37.3	63	30.6	72	35.6	70	33.3	
									0.51
Smoking status									
Never	115	53.4	118	55.4	115	56.4	138	64.2	
Former	81	37.7	78	36.6	72	35.3	67	31.2	
Current	19	8.8	17	8.0	17	8.3	10	4.7	
									0.31
Recent alcohol intake									
No	45	22.6	54	28.0	56	29.6	56	28.0	
Yes	154	77.4	139	72.0	133	70.4	144	72.0	
Family history of breast cancer									
No	169	81.3	180	86.1	168	83.2	176	83.0	
Yes	39	18.8	29	13.9	34	16.8	36	17.0	
									0.60
Hormone use									
Never	61	28.6	51	23.9	55	27.1	46	21.6	
Former	32	15.0	33	15.5	24	11.8	36	16.9	
Current	120	56.3	129	60.6	124	61.1	131	61.5	
									0.54

^a^Missing values for cases and controls, respectively, were as follows: body mass index (1, 1), age at menarche (1, 0), number of livebirths (0, 2), age at first birth (0, 3), personal history of benign breast disease (11, 6), alcohol intake (35,31), family history of breast cancer (8, 8), and hormone use (2, 3). *Q* quartile

As shown in Table 3, odds ratios and 95% CI, adjusted for age and time of entry and period of DNA extraction, were 1.0 (referent), 0.89 (95% CI, 0.6–1.3), 0.88 (95% CI, 0.6–1.3), and 0.84 (95% CI, 0.6–1.2) comparing women in the highest to the lowest batch-specific quartile levels of %5-mdC (*p* for trend = .39). Results were essentially unchanged after additional adjustment for breast cancer risk factors (*p* for trend = .35). When we restricted analyses to 298 cases who were hormone-receptor-positive (estrogen-receptor-positive and progesterone-receptor-positive) and 419 controls, comparable fully-adjusted odds ratios and 95% CI were similar: 1.0, 0.82 (0.5–1.3), 0.98 (0.6–1.5), and 0.85 (0.5–1.3) (data not shown).

**Table 3:** Quartile level of WBC DNA methylation and risk of breast cancer

	Quartiles of DNA methylation			
	Q4 (high)	Q3	Q2	Q1 (low)
Adjusted OR^a^	1.0 (referent)	0.89	0.88	0.84
95% CI		(0.6–1.3)	(0.6–1.3)	(0.6–1.2)
Cases, controls	114, 101	107, 106	102, 102	105, 110
Fully adjusted OR^b^	1.0 (referent)	0.90	0.87	0.83
95% CI		(0.6–1.3)	(0.6–1.3)	(0.6–1.2)
Cases, controls	114, 101	107, 106	102, 102	105, 110

*WBC* white blood cells, *Q* quartile, *OR* Odds Ratio

^a^Adjusted for age, time of entry and date of DNA extraction (previously extracted, newly extracted)

^b^Additionally adjusted for race, body mass index, age at menarche, age at first live birth, number of children, type of menopause, age at natural menopause, personal history of benign breast disease, cigarette smoking, alcohol intake, family history of breast cancer, and hormone use

In further analyses, we stratified on period of DNA extraction (Table 4). Among the group with previously extracted DNA, we observed a nonsignificant increase (OR = 1.44, 95% CI, 0.8–2.7) in risk in the lowest quartile of %5-mdC compared to those in highest quartile. In the group with newly extracted DNA, however, there was an unexpected decreasing trend in the ORs from the highest to lowest quartile of %5-mdC (*p* for trend = .05). Risk was also significantly decreased in the lowest quartile of %5-mdC compared to those in the highest quartile (OR = 0.61, 95% CI, 0.40–1.0).

**Table 4:** Quartile level of WBC DNA methylation and risk of breast cancer by date of DNA extraction, and by time between blood collection and diagnosis, stratified by DNA extraction method

	Quartiles of DNA methylation			
	Q4 (high)	Q3	Q2	Q1 (low)
Period of DNA extraction				
Previously extracted DNA				
Adjusted OR^a^	1. 0 (referent)	0.99	1.01	1.44
95% CI		(0.5–1.9)	(0.5–2.0)	(0.8–2.7)
Cases, controls	33, 36	33, 36	34, 36	51, 39
Newly extracted DNA				
Adjusted OR^a^	1.0 (referent)	0.84	0.82	0.61
95% CI		(0.5–1.3)	(0.5–1.3)	(0.4–1.0)
Cases, controls	81, 65	74, 70	68, 66	54, 71
Time from blood collection to diagnosis				
Previously extracted DNA				
1 to <2 years				
Adjusted OR^a,b^	1.0 (referent)	0.99	1.16	1.41
95% CI		(0.4–2.7)	(0.4–3.0)	(0.6–3.6)
Cases, controls	11, 36	10, 36	13, 36	16, 39
2 to <4 years				
Adjusted OR^a,b^	1.0 (referent)	1.15	1.00	1.39
95% CI		(0.5–2.7)	(0.4–2.4)	(0.6–3.2)
Cases, controls	14, 36	16, 36	14, 36	21, 39
≥ 4 years				
Adjusted OR^a,b^	1.0 (referent)	0.89	0.97	1.53
95% CI		(0.3–2.8)	(0.3–3.1)	(0.6–4.3)
Cases, controls	8, 36	7, 36	7, 36	14, 39
Newly extracted DNA				
2 to <4 years				
Adjusted OR^a,b^	1. 0 (referent)	0.88	0.73	0.68
95% CI		(0.5–1.5)	(0.4–1.3)	(0.4–1.2)
Cases, controls	43, 65	43, 70	32, 66	33, 71
≥4 years				
Adjusted OR^a,b^	1.0 (referent)	0.80	1.00	0.51
95% CI		(0.4–1.5)	(0.6–1.8)	(0.3–1.0)
Cases, controls	38, 65	31, 70	36, 66	21, 71

^a^Adjusted for age and time at entry

^b^Same controls are used for each category of elapsed time from blood collection to diagnosis, within period of DNA extraction (previously extracted, newly extracted); *OR* Odds Ratio

We further stratified on both period of DNA extraction and elapsed time from blood collection to diagnosis. Inherent to the study design, all of the breast cancer cases diagnosed within 1- < 2 years of blood collection had previously extracted DNA. When we restricted the analysis to study subjects with previously extracted DNA, we observed nonsignificant slight increases in risk in the lowest quartile level of %5-mdC in all three categories of years since blood collection (i.e. 1- < 2, 2- < 4, ≥4 years). When we restricted analysis to study subjects with newly extracted DNA, however, we found no evidence of any increased risk in women in either the 2- < 4, or ≥4 years since blood collection.

## Discussion

Overall, we found no evidence that lower levels of %5-mdC in white blood cell DNA were associated with increased breast cancer risk in a case-control study nested in the PLCO cohort. The %5mdC assay provides a comprehensive measure of genome-wide DNA methylation and is considered the gold standard for accuracy. Odds ratio (OR) estimates and 95% confidence intervals (CI) for breast cancer risk adjusted for age at entry, year of entry, and period of DNA extraction, were 1.0 (referent), 0.89 (95% CI, 0.6–1.3), 0.88 (95% CI, 0.6–1.3), and 0.84 (95% CI, 0.6–1.2) for women in the highest compared to lowest quartile levels of %5md-C (*p* for trend = .39). There was some variability in our results depending on whether the DNA was previously or newly extracted. We observed a nonsignificant increased risk in the lowest quartile of %5mdC in the subset of women with previously extracted DNA, whereas risk was significantly decreased in the lowest quartile of %5mdC in the subset of women with newly extracted DNA. One possibility is that this difference in the results by period of DNA extraction is the result of technical issues or sample degradation. Conceivably, the earlier method of extraction or shorter buffy coat storage of the previously extracted samples may have resulted in less non-differential misclassification in our methylation measure. In a recent reliability study of methylation measures from mononuclear cells using the HumanMethylation450K Bead Array, differences in DNA extraction methods (and possible differences in cell composition resulting from them) were suggested to have contributed to lower observed reliability for repeated samples across studies than for technical replicates within a study [26]. Given that our findings that previously and newly extracted DNA were in opposing directions and that there was a larger sample size of newly extracted DNA, chance due to small numbers after stratification is a likely explanation for the observed variability.

Our results are not consistent with those of the one other breast cancer study to measure global DNA hypomethylation with %5-mdC, a small case-control study in which WBC DNA was collected after breast cancer diagnosis [11]. In that study, risk of breast cancer was nearly three times higher among those with in the lowest tertile of %5-mdC compared to those in the highest tertile. A major strength of our study is that we purposely included only breast cancer cases in which DNA was collected at least 12 months prior to diagnosis. We found no evidence that risk varied according to time elapsed between blood collection and breast cancer diagnosis.

As well-summarized in a recent systematic review [27], the weight of evidence also does not support an association between breast cancer risk and WBC DNA methylation, measured in other studies by surrogate marker methods, such as *Alu*, *LINE*-*1*, or LUMA. Of seven retrospective case-control studies [11, 14–19] and four studies with prospectively collected pre-diagnostic WBC DNA (results from three cohorts were presented in a single publication) [12, 13] that measured global methylation by *Alu* and *LINE*-*1* methylation, only one [12] observed a significantly higher risk of breast cancer among those who had lower LINE-1 methylation levels. Three retrospective case-control studies have examined the relation between WBC DNA global methylation levels and breast cancer risk using LUMA, with inconsistent results [15, 20, 21]. As suggested by Brennan and colleages [22], LINE-1 and other surrogate assays are likely not sufficiently sensitive to detect slight inter-individual differences in WBC DNA methylation. Indeed, Brennan and colleagues reported that the population variability in WBC DNA *LINE*-*1* methylation measured by pyrosequencing in prospectively collected blood did not statistically exceed that of technical duplicates [13]. Further, Tang and colleagues [27] noted that the findings from WBC DNA methylation studies that have evaluated *LINE*-*1* and breast cancer have been null with one exception, despite using different methods of detection (e.g., combined bisulfite restriction analysis, pyrosequencing, MethylLight). Interestingly, a particular strength noted of the prospective study that detected a statistical association between WBC DNA *LINE*-*1* hypomethylation and breast cancer risk was that it employed three independent bisulfite conversions, PCR, and pyrosequencing reactions on each sample [12]. In our prospectively collected blood specimens, we also observed limited population variability in %5mdC levels in WBC DNA, adding to concern that even a small amount of laboratory error is problematic in studies that involve quantification of global WBC DNA methylation from healthy individuals.

Several recent studies have estimated global methylation by averaging individual CpG site-specific methylation levels over the hundreds of thousands of CpG sites on the Illumina HumanMethylation450 BeadChip [7, 8]. This alternative approach measures less than 5% of the 28 million CpG sites in the genome [28]. In two prospective cohort analyses that used this approach to measure WBC DNA methylation, women in the highest quartile of methylation had about a 50% decrease in risk of breast cancer compared to women in the lowest quartile of methylation [7, 8]. Another cohort analysis, which used pooled samples and next-generation sequencing of the overlapping CpG sites from the Illumina HumanMethylation450 BeadChip, also found higher levels of genome-wide methylation in controls than in cases [7]. However, findings from a fourth cohort study were null [7]. Subanalyses based on genomic region have been inconsistent. One study found that WBC epigenome-wide methylation in the promoter region was associated with an increase in breast cancer risk whereas epigenome-wide methylation outside the promoter region was associated with a decreased risk of breast cancer [8]. A second study confirmed that WBC epigenome-wide methylation in gene bodies was associated with decreased risk but was unable to replicate the increase in risk with promoter region methylation [7]. These findings may need to be interpreted with caution given a recent report that measurement error and limited variability in DNA methylation measures from mononuclear cells is problematic for a substantial proportion of of CpG sites on the HumanMethylation450 BeadChip [26].

Another potential limitation of our study is that we studied %5-mdC in a composite of DNA from different types of white blood cells. As previously noted by others [29, 30], the WBC distribution can vary across individuals and the level of global DNA methylation can vary by cell type. The study by Choi and colleagues also used composite DNA [11] as have nearly all other prior WBC methylation studies because it is simple and yields the most DNA [30]. Another potential limitation of our study is the batch-to-batch variation in %5mdC that we observed. This issue necessitated creating quartile cut points for %5mdC separately for individuals within each laboratory batch, a method also employed in the case-control study by Choi and colleagues [11].

## Conclusions

In summary, our study, as well as the published literature, which has used a range of methods for assessing global methylation, including Alu, LINE-1, LUMA, and %5-mdC assays, does not support a link between WBC DNA methylation and breast cancer risk. It is conceivable a more accurate and precise measure of global methylation is needed. However, if there is an association between WBC DNA methylation patterns and breast cancer risk, it is likely that the at-risk methylation pattern is more complex and restricted to specific regions and will need to be identified through measures of sequence-specific or gene-specific DNA methylation [13, 31].

## Abbreviations

%5-mdC: Ratio of 5-methyl-2’ deoxycytidine (5-mdC) to 2’-deoxyguanine (dG)
CCGG: Cytosine-cytosine-guanine-guanine sequence
CI: Confidence interval
CpG: Cytosine-phosphate-guanine dinucleotide
LC-ESI-MS/MS: Liquid chromatography-electrospray ionization-tandem mass spectrometry
LINE-1: Long interspersed nuclear element 1
LUMA: Luminometric methylation assay
m/z: Mass-to-charge ratio
OR: Odds ratio
PCR: Polymerase chain reaction
PLCO: Prostate, Lung, Colorectal, and Ovarian Cancer Screening Trial
WBC: White blood cell
